# The Norfolk and Norwich Hospital

**Published:** 1922-05

**Authors:** Frank Inch

**Affiliations:** Secretary, Norfolk and Norwich Hospital


### CORRESPONDENCE.

[Correspondence on all subjects is invited, but we cannot in any way be responsible for the opinions expressed by our correspondents, who must give name and address as a guarantee of good faith, but not necessarily for publication. Correspondents are reminded that conciseness greatly facilitates early insertion.]

### The Norfolk and Norwich Hospital.

*To the Editor of* THE HOSPITAL AND HEALTH REVIEW.

SIR,—The paragraph in your March issue, headed "The Norwich Provident Scheme," is not correct, and I should be obliged if you would insert a note to this effect in your next issue. We have no "Provident Scheme" on the lines referred to by you in connection with this hospital, and your report only referred to a scheme which was proposed at a meeting in a neighbouring town, but which could not be accepted by this hospital. The reason of the hospital's inability to accept such a provident scheme is due to the fact that we have had in operation for the last 18 months a contributory scheme which has proved wonderfully successful. Under this scheme contributors of not less than 1d. per week (and their dependents) are exempt from all hospital payments, and from this source the Norfolk and Norwich Hospital received £11,280 in the year 1921.

The success of this contributory scheme accounted, to a large extent, for the very successful financial position last year. In 1919 our expenditure exceeded our income by £10,601 and, in 1920, by £13,591, but last year the income exceeded the expenditure by £7,059. By the continued progress of the contributory scheme the serious financial difficulties of the hospital at the end of 1920 are gradually being removed.

Yours faithfully,
FRANK INCH,
*Secretary, Norfolk and Norwich Hospital.*

[The meeting at Attleborough referred to in our columns ended with a resolution, unanimously carried, that "a scheme should be organised, &c.," and the scheme was therefore described as "started." No doubt, the hospital's decision, mentioned by Mr. Inch, not to accept the scheme, has altered the position; and it is gratifying to learn that the hospital has already benefited so handsomely from contributions.—ED.]

### Hospital Officials' Pensions.

*To the Editor of* THE HOSPITAL & HEALTH REVIEW.

